# Surgical treatment of CRPS

**DOI:** 10.1186/1753-6561-9-S3-A83

**Published:** 2015-05-19

**Authors:** Igor Golubev

**Affiliations:** 1 Priorov Central Institute of Trauma and Orthopedics, Moscow, Russia

## 

We investigated 82 cases of upper extremity trauma which were complicated by CRPS. All of them were evaluated by thermography and laser Doppler flourometry. The cases of sympathetically dependent and independent forms of CRPS were divided. In the 49 cases of sympathetically dependent form, thoracoscopic sympathectomy was performed in 33 and paravasal sympathectomy in 16 cases. Mean VAS pain scores before the procedure were 7.8 and 7.2 respectively and two weeks after were 0.9 and 1.2 respectively. There was also a significant amount of decrease in swelling and increasing of range of joint motion noted.

